# Repair of Giant Parastomal Hernia with Loss of Domain by Total Colectomy: A Case Report

**DOI:** 10.70352/scrj.cr.25-0002

**Published:** 2025-07-23

**Authors:** Tomoaki Kaneko, Mitsunori Ushigome, Satoru Kagami, Kimihiko Yoshida, Yasuyuki Miura, Takayuki Suzuki, Akiharu Kurihara, Nagato Shimada, Kimihiko Funahashi

**Affiliations:** 1 Department of Gastroenterological Surgery, Toho University Medical Center Omori Hospital, Tokyo, Japan; 2 Department of Surgery, Sagamihara Central Hospital, Sagamihara, Kanagawa, Japan

**Keywords:** loss of domain, parastomal hernia, total colectomy, incisional hernia repair

## Abstract

**INTRODUCTION:**

Parastomal hernias with loss of domain are those in which it is difficult to return the hernia contents to the abdominal cavity and close the hernia orifice using standard mesh repair methods. We encountered a case in which abdominal wall closure was achieved safely by reducing the hernia content through bowel resection.

**CASE PRESENTATION:**

The patient, a 50-year-old man, had a history of ulcerative colitis with a complex anal fistula, resulting in construction of a stoma with double orifices using the sigmoid colon. He presented requesting surgery because his parastomal hernia had increased greatly in size, and stoma management became difficult. Abdominal CT showed a large hernia with an incisional hernia volume to peritoneal volume ratio >80%. Total colectomy was performed, and a stoma was reconstructed at another site. The hernia orifice was closed using a fascia lata graft. No postoperative abdominal compartment syndrome was observed. Six months later, abdominal CT showed a small hernia of the abdominal wall; however, the stoma was easily managed, and the patient’s quality of life improved.

**CONCLUSIONS:**

Bowel resection for volume reduction may be an effective option for the repair of incisional hernias with loss of domain.

## Abbreviations

ACS
: abdominal compartment syndrome
BMI
: body mass index
IHV/PV
: incisional hernia volume/peritoneal volume
PPP
: progressive preoperative pneumoperitoneum

## INTRODUCTION

Loss of domain is defined as, “a ventral hernia large enough such that simple reduction in its contents and primary fascial closure either cannot be achieved without additional reconstructive techniques or cannot be achieved without significant risk of complications due to the raised intra-abdominal pressure”.^1)^ The most common surgical procedure for repair of parastomal hernias is to reduce the hernia contents into the abdominal cavity and close the hernia orifice using mesh. However, when loss of domain occurs, it is difficult to reduce the large amount of hernia contents back into the abdominal cavity. Also, the relocation of a deformed stoma is often necessary, which makes hernia treatment difficult using standard methods. Herein, we report a case in which a large parastomal hernia was treated with total colectomy, enabling safe abdominal wall closure with a fascia lata graft.

## CASE PRESENTATION

A 50-year-old man underwent the creation of a stoma with double orifices in the sigmoid colon via an intraperitoneal route for ulcerative colitis and a complex anal fistula 16 years prior. Nine years previously, he developed a parastomal hernia but was kept under observation because of multiple comorbidities. However, the hernia gradually grew larger, making it difficult to manage the stoma; therefore, he presented for consideration of a hernia repair.

The patient lived with his brother who cooked most of his meals, making it difficult for him to strictly manage his diet. Additionally, hemiparesis secondary to cerebral infarction limited his ability to participate in adequate physical therapy. Inpatient management was also challenging because our hospital lacks an obesity treatment specialist, and his history of schizophrenia made it difficult to find another facility willing to accept him. Furthermore, continuation of treatment after discharge was anticipated to be difficult, and was therefore discontinued. His medical history included ulcerative colitis, schizophrenia, cerebral and pulmonary infarctions, deep vein thrombosis, and hypertension.

The patient had been receiving salazosulfapyridine for ulcerative colitis since disease onset 28 years prior, up until hernia surgery. Endoscopic follow-up had not been performed in recent years due to stoma deformity and the development of a cerebral infarction. However, endoscopic findings from 6 years earlier were consistent with clinical remission.

**Fig. 1:**
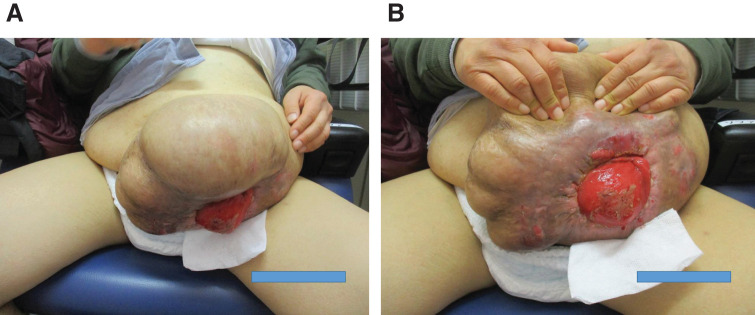
(**A**, **B**) Patient presentation. A colostomy with double orifices was placed in the left lower abdomen and a huge parastomal hernia is observed. Stoma management was difficult due to the distended abdominal wall.

Physical examination revealed that the patient was obese with a height of 167 cm, weight of 85 kg, and BMI of 30.4. In addition, the patient had incomplete right-sided paralysis due to the cerebral infarction. A large parastomal hernia was observed in the left lower abdomen, and the skin around the stoma was thin and eroded (**Fig. 1A** and **1B**).

**Fig. 2:**
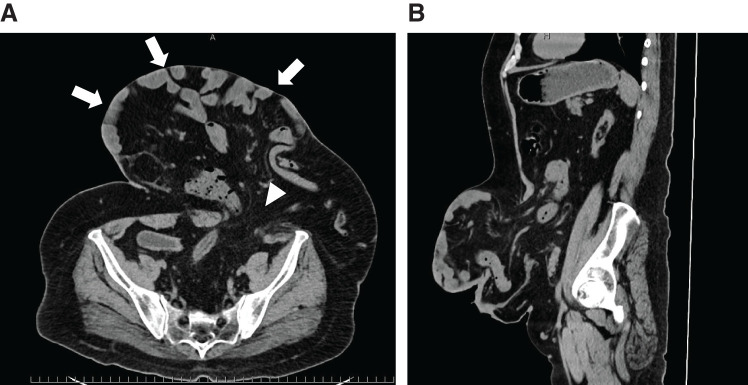
(**A**, **B**) CT findings. CT shows a giant hernia around the stoma with contents of the colon and small intestine(➡). The hernia portal is approximately 9 × 9 cm (▲).

Blood analysis showed mild hypoalbuminemia (3.5g/dl) and pulmonary function tests revealed a restrictive lung disorder, with a vital capacity of 2.82 L (68.6%). Plain abdominal CT revealed a giant hernia containing the colon and small intestine around the stoma (**Fig. 2A** and **2B**). The hernia orifice measured 9 cm × 9 cm. Calculations using the Synapse Vincent system version 6.4 (Fujifilm, Tokyo, Japan) yielded a volume of hernia contents/volume of abdominal cavity contents of 6066.1 mL/7246.4 mL (83.7%). Defecography performed 2.5 years ago showed decreased anal function, making stoma closure impossible.

The surgical indication and approach were discussed, and consensus was reached among the wound ostomy care nurse, colorectal surgeons, and hernia surgeons. Perioperative management was planned in collaboration with anesthesiologists and intensive care unit physicians. Based on these discussions, a detailed explanation of the risks and benefits of surgery was provided to the patient and his brother, and informed consent was obtained. Given the patient’s psychiatric condition, particular attention was given to ensure the brother’s comprehension through multiple individualized discussions.

**Fig. 3:**
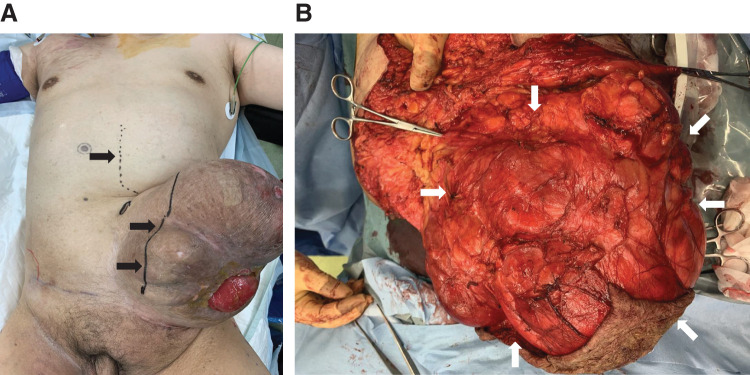
Incision and intraoperative findings. (**A**) The upper abdomen with a skin incision made in the midline. In the lower abdomen, the fragile skin around the stoma hernia is excised (➡). (**B**) Hernia contents (large intestine and small intestine) are found enclosed in a hernia sac around the stoma (➡) and cannot be reduced into the abdominal cavity, even under general anesthesia.

During surgery, a skin incision was made from the midline of the upper abdomen to trim the weakened skin around the stomal hernia (**Fig. 3A**).

The small and large intestines, wrapped in hernia sacs, prolapsed around the stoma and could not be reduced even under general anesthesia (**Fig. 3B**).

First, the colon was passively mobilized from the stoma toward the oral side, and the intestine was transected at the level of the descending colon using a linear stapler (Signia Stapling System; Medtronic, Minneapolis, MN, USA). Next, moving to the right side, the terminal ileum was dissected using a linear stapler, and the colon was mobilized clockwise from the ascending to the descending colon and then mesenterized.

**Fig. 4:**
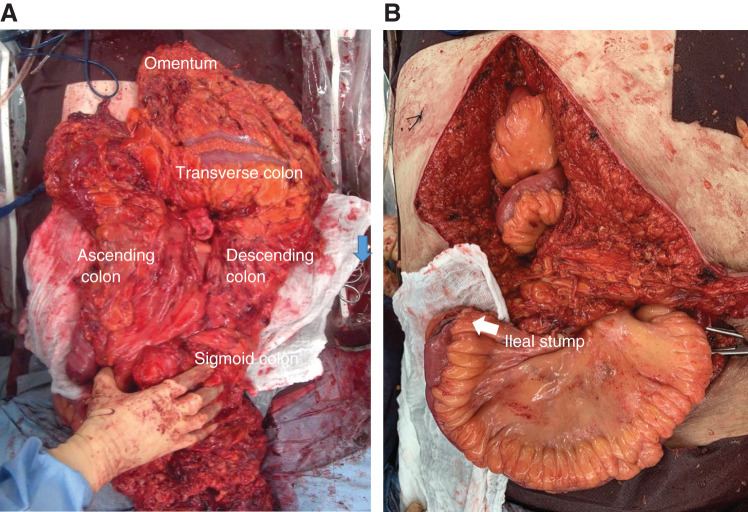
Dissection and removal of colon, and creation of ileostomy. (**A**) The large intestine (from the cecum to the sigmoid colon) including the stoma, part of the rectum (Rs), and the omentum are removed. (**B**) After the resection, abdominal wall closure is possible without tension. An endostoma is created in the right upper abdomen using the ileal stump.

**Fig. 5:**
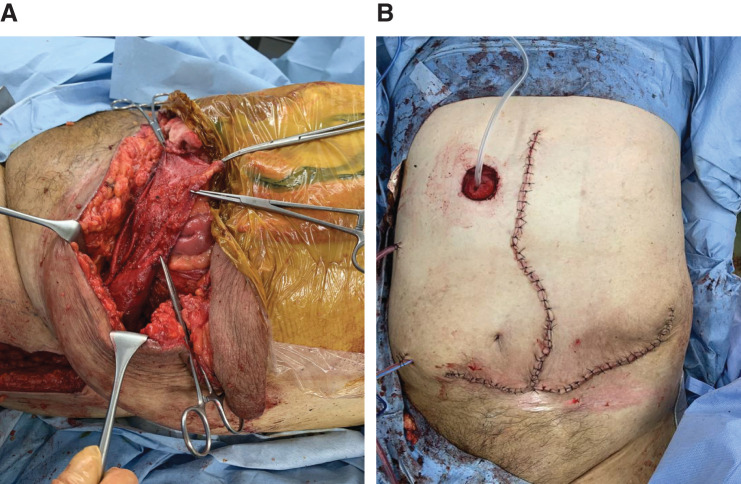
Closure of abdomen. (**A**) The hernia orifice is closed using a left fascia lata flap. (**B**) Excess skin is removed, and the wound is closed.

The mesenteric artery was ligated near the root and the mesentery was resected as much as possible. The omentum was also completely resected from the gastroepiploic artery to the transverse colon. The remaining sigmoid colon was then mobilized, and the rectum was dissected and removed at the level of the promontorium using a linear stapler (**Fig. 4A**). An end ileostomy was performed in the right upper abdomen (**Fig. 4B**). The hernia orifice was closed with a left fascia lata graft, and the midline wound was closed in two layers without using a mesh. The excess skin was removed (**Fig. 5A** and **5B**).

The operation time was 11 h 22 min, and the blood loss was 1758 mL. The histopathological findings indicated that the patient had low-activity ulcerative colitis. Due to the high risk of postoperative complications, the patient was admitted to the intensive care unit and managed with a ventilator. Muscle relaxants were administered until the 1st POD, sedatives until the 4th POD, and the patient was extubated on the 12th POD. Intraperitoneal pressure was measured 4 times a day until the 4th day after the operation and was 5.1–13.08 mmHg, with no signs of ACS. However, he developed acute kidney injury and was managed with temporary hemodialysis. After 15 days of ICU management, the patient was transferred to a general ward and then to another hospital for rehabilitation at the 62nd POD.

**Fig. 6:**
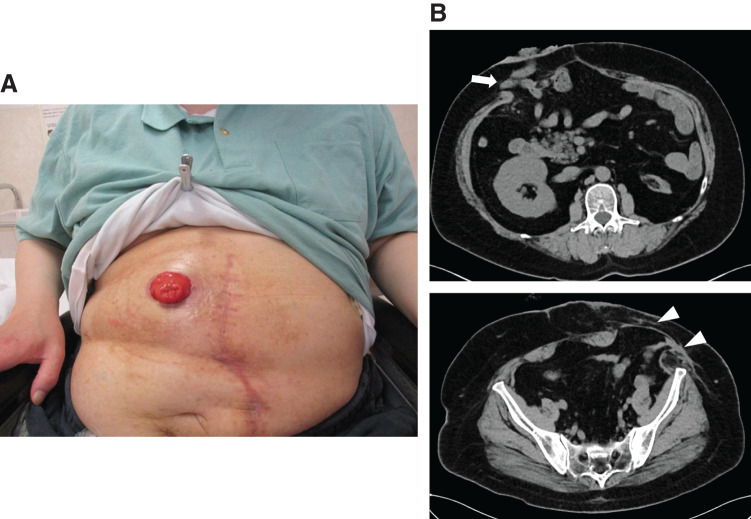
Follow-up. (**A**) A physical examination 6 months later shows that the abdominal wall is flat, making stoma management easier. (**B**) A plain abdominal CT shows a small hernia (➡) at the stoma site, but no hernia at the former stoma site (▲).

Physical examination 6 months later revealed that the abdomen was flat, and stoma management proceeded without difficulty. No diarrhea or electrolyte abnormalities related to the ileostomy were observed. Abdominal CT revealed a small parastomal hernia at the site of reconstruction; however, there was no recurrence at the closed stoma site (**Fig. 6**).

## DISCUSSION

In hernias with loss of domain, the fascia can be repaired without tension when the IHV to PV ratio is less than 20%.^2)^ In this case, the IHV/PV ratio was over 80%, making it difficult to return the hernia contents to the abdominal cavity and close the abdominal wall, and markedly increasing the risk of postoperative ACS.

PPP and botulinum toxin injections have been reported as methods to reduce the risk of ACS before surgery. In PPP, gases such as oxygen or nitrous oxide are periodically injected into the abdominal cavity through a Veress needle or an indwelling intraperitoneal catheter to expand the abdominal wall.^3)^ On the other hand, Botulinum toxin A can be injected into the lateral abdominal muscle to cause paralysis, thereby expanding the abdominal wall.^4)^ The combination of these 2 techniques can reduce the IHV/PV by a reported average of 14%,^5)^ but this was not considered sufficient in this case. Additionally, these procedures are not covered by health insurance in Japan.

There are methods to extend the abdominal wall during surgery, such as releasing the external oblique muscle^6)^ or the transverse abdominis muscle,^7)^ which can be used to elongate the abdominal wall by up to 20 cm at the waistline.^8,9)^ In the case of parastomal hernia, however, the presence of a stoma makes it impossible to achieve effective abdominal wall advancement on both sides. In addition, due to the large amount of prolapsed hernia contents and the size of the hernia orifice, it seemed difficult to avoid ACS after repairing the hernia, even if we used these methods. Therefore, we performed a total colectomy to reduce the hernia contents, as the patient’s long-standing history of ulcerative colitis was a high-risk factor for colorectal cancer.^10)^ Because the hernia orifice was large, and the stoma was severely deformed, the stoma was relocated.

Although its mechanical strength is limited, a fascia lata graft was used for stoma closure because it resists infection and can cover a large defect without requiring abdominal wall advancement.^11)^

Only one case series has described the use of intestinal resection to repair abdominal wall hernias.^12)^

This report included 11 patients with a mean BMI of 43 (23–52) kg/m^2^ and a mean volume ratio of hernia volume to abdominal cavity volume of 70 (48–100)%. PPP was performed in 82% (9/11) of patients, and right hemicolectomy + mesh placement was performed in all cases. Bariatric surgery was also performed in 27% (3/11) of the patients. Recurrence occurred in 73% (8/11) of the patients after the initial surgery, but half of the patients underwent reoperation. After a follow-up of 27 ± 18 months, the hernia residual rate was 45% (5/11), and the authors concluded that this was an effective salvage surgery.

In our case, surgery facilitated stoma management and improved the patient’s quality of life. However, several issues were associated with performing surgery. First, it was not possible to predict before surgery the amount of the intestine that would need to be resected to safely close the abdominal wall.

In a report by Benoit et al.,^12)^ only 1 of 11 cases with peritonitis due to anastomotic leakage developed ACS. This finding suggests that a right hemicolectomy was sufficient for safe abdominal wall closure in many of their cases. In cases where right hemicolectomy is insufficient to reduce the volume of the hernia contents, we believe that total colectomy will enable abdominal wall closure in almost all cases.

However, few studies have investigated the use of intestinal resection for giant hernia repair, and data supporting its safety and efficacy for this indication are lacking.

In addition, the parameters determining whether the abdominal wall can be closed safely include not only the resection volume but also the elasticity of the abdominal wall muscles and mesentery, and the mobility of the diaphragm.^12)^ Accumulation of data including these factors is required to predict whether right hemicolectomy will be sufficient, or whether total colectomy will be required for abdominal wall closure.

In our case, no ileostomy-induced dehydration or electrolyte abnormalities were observed after total colectomy, which would have otherwise necessitated antidiarrheal measures such as loperamide hydrochloride.^13)^ Similarly, an adequately performed right hemicolectomy can effectively prevent diarrhea.

Another concern is that intestinal resection increases the risk of mesh infection.^14,15)^ In this study, we used a plastic surgical procedure without mesh to avoid infection risk. However, some reports suggest that intestinal resection does not increase the risk of mesh infection.^16)^ Similarly, mesh is effective in shortening the operation time and preventing recurrence from stoma closure^17)^ and creation.^18)^ The feasibility of using mesh is a topic for future studies.

Finally, in this case, a parastomal hernia developed at the newly created stoma site. Parastomal hernias have a high recurrence rate even after surgical repair; therefore, conservative management through observation is typically adopted in asymptomatic patients or those with multiple comorbidities.^19,20)^ As hernias are benign conditions, actively recommending surgery is challenging when patients decline the procedure or when surgical risk is high. However, observation carries risks such as incarceration and hernia progression, which increase the technical difficulty of future surgery.^20)^ In this case, prolonged observation resulted in a “loss of domain,” a condition that requires further investigation. At present, the patient is asymptomatic and declines surgery; thus, observation is continued. Nevertheless, should symptoms develop and impair the quality of life, we plan to perform surgical repair using mesh before the condition becomes technically challenging.

## CONCLUSIONS

Large intestinal resection may be an effective option for abdominal incisional hernias with loss of domain.

## DECLARATIONS

### Funding

This study received no specific grants from any funding agency in the public, commercial, or not-for-profit sector.

### Authors’ contributions

T.K. contributed to the design of the study and wrote the draft manuscript.

M.U. participated in the data interpretation and contributed to the discussion.

S.K. contributed to the acquisition of data and discussion.

K.Y. and Y.M. participated in the surgical procedures and contributed to the discussion.

T.S. and A.K. contributed to the acquisition of data and discussion.

N.S. supervised the study and critically revised the manuscript.

K. F. participated in the surgical procedures and contributed to the design of the study.

All the authors have read and approved the final version of the manuscript.

### Availability of data and materials

Not applicable.

### Ethics approval and consent to participate

This work does not require ethical considerations or approval. Written informed consent was obtained from the patient.

### Consent for publication

Written informed consent was obtained from the patient for the publication of this case report.

### Competing interests

The authors disclose no conflicts of interest.
